# Heat Transfer, Molten Pool Flow Micro-Simulation, and Experimental Research on Molybdenum Alloys Fabricated via Selective Laser Melting

**DOI:** 10.3390/ma14010075

**Published:** 2020-12-25

**Authors:** Zhenping Guo, Lei Wang, Cheng Wang, Xiangyu Ding, Jichao Liu

**Affiliations:** 1 Basic Department, Air Force Engineering University, Xi’an 710000, China; pluto_70@163.com (Z.G.); warrant_74@126.com (C.W.); L_JiChao@163.com (J.L.); 2 College of Aircraft Engineering, Nanchang Hangkong University, Nanchang 330000, China; dxiangyu20000@163.com

**Keywords:** molybdenum alloys, selective laser melting, spreading/solidification time

## Abstract

Molybdenum-based alloys fabricated via selective laser melting are considered to represent the next generation of high-temperature structural materials, but the additive manufacturing technology aiming at refractory alloys has not been explored extensively. Multi-field coupling simulation can be used as a practical tool to simulate a single track of molybdenum alloy printed via selective laser melting, observe the topography of the molten pool over time, and determine the effect of Marangoni flow on defect suppression. In this study, the t_melt_, t_vapor_, and the competition mechanism of spreading/solidification time were considered, the dominant spreading time was calculated, and a reasonable process parameter window for fabricating molybdenum alloy was obtained. It was found that keeping the energy density in the range of 3.1 × 10^11^ J/m^3^–4.0 × 10^11^ J/m^3^ could better maintain appropriate melt channel depth and width and was beneficial to the droplet spreading behavior. This range was deemed suitable for printing molybdenum alloy.

## 1. Introduction

The aero engine is a critical component that determines the performance of the aircraft. With regard to engine parts, the superalloy is a significant material in the hot end component of the aero engine. The usage of the superalloy accounts for 55–65% of the total weight of the aero engine. Turbine blades made of nickel-based alloys can be operated at approximately 1150 °C, which is close to 90% of its melting point [1], and do not meet the needs of ultra-high temperature performance. At present, Mo-Si, Nb-Si, and Si-C ceramic matrix composites are the main candidates for use in the high-temperature parts of aero engines in the future [2]. The specific strength of Si-C ceramic matrix composites is low, and their intrinsic brittleness is too great. They are not suitable for load-bearing parts with complex shapes at high temperature, and it is difficult to connect ceramic matrix composites with metal materials. The specific strength and oxidation resistance of Nb-Si alloy above 1500 K are clearly weaker than for the Mo-Si alloy. Therefore, development of the Mo-Si alloy will play an important role in the application of superalloys. Refractory molybdenum can be used as the matrix and solid solution strengthening elements can be added to form a high-temperature material strengthened by the precipitation phase. The melting point of the molybdenum-based superalloy is up to 2270 K, and it is considered a refractory superalloy [3,4,5]. Its continuous Mo matrix can provide strong mechanical properties. The recently proposed molybdenum silicon boron alloy processed by selective laser melting (SLM) is expected to become part of the next generation of superalloys, replacing nickel-based alloys [6,7]. Molybdenum silicon boron alloy exhibits excellent room temperature toughness and high temperature oxidation resistance, and the T1(Mo_5_SiB_2_) phase produced by the non-equilibrium state process of selective laser melting can show better high-temperature creep resistance [3].

Some reports have explored the formation of such high-temperature refractory alloys. Wang et al. fabricated a dense and crack-free pure Mo alloy via laser melting, and found that cracks can be suppressed by designed supporting structure [8]. Maamoun et al. studied the interaction between SLM process parameters by analyzing relative density, porosity, surface roughness, and dimensional accuracy [9]. Saprykin et al. discussed the influence of parameters such as light output power, laser moving speed, and preheating temperature on the formation mechanism of solidified particles during powder melting [10]. Furthermore, in recent years, many studies also have reported on laser powder bed fusion simulation. Khairallah studied the micro-mechanism in the simulation of molten pool flow and found that evaporative cooling is an important factor affecting the peak temperature [11]. Zhang revealed that the depth and length of the molten pool, porosity and grain density depended on process parameters via simulation [12]. In addition, Bai et al. reported that pores and defects were the main reasons for low density of the components with high melting points [13]. Kruth et al. reported that if the droplet was not completely spread out, it would cause the occurrence of spheroidization defects [14]. However, previous studies lack simulation experiments on high-temperature refractory alloys, and there is almost no research on the time characterization window from the droplet propagation behavior. Therefore, for pure molybdenum, a material with a high melting point and high thermal conductivity, it is necessary to prevent the droplet from solidifying before it is fully expanded, promote the wetting behavior, and ensure sufficient spreading time. At the same time, porosity reduction and defect suppression are also actual concerns for processing refractory alloys. Hence, it is necessary to expand the adaptability of the SLM method in the processing of Mo-based superalloys and accelerate the application of Mo-based alloys (such as molybdenum silicon boron alloys) in the aerospace field. Multi-field simulation as a practical tool can be used to simulate the SLM processing of refractory molybdenum alloys and reduce experimental workload.

Consequently, the objective of this paper was to explore a suitable processing window and find reasonable explanations via the analysis of a single-melt channel simulation and actual experiment, the study of micro-melt pool morphology and the laser interaction time for forming refractory molybdenum alloy.

## 2. Model Approaches

### 2.1. Heat Transfer and Molten Pool Flow Control Equations

Based on the above basic assumptions, the governing equations of the mass continuity equation, momentum and energy equation are given here.

Mass continuity equation:

(1) $$\frac{\partial \rho }{\partial t}+\nabla •(\rho V)=0$$

Momentum conservation equation:

(2) $$\frac{\partial (\rho u)}{\partial t}+\nabla •(\rho Vu)=\nabla (\mu \nabla u)-\frac{\partial p}{\partial x}+S_{u}$$

(3) $$\frac{\partial (\rho v)}{\partial t}+\nabla •(\rho Vv)=\nabla (\mu \nabla v)-\frac{\partial p}{\partial y}+S_{v}$$

(4) $$\frac{\partial (\rho w)}{\partial t}+\nabla •(\rho Vw)=\nabla (\mu \nabla w)-\frac{\partial p}{\partial z}+S_{w}$$

Energy conservation equation:

(5) $$\frac{\partial (\rho T)}{\partial t}+\nabla (\rho VT)=\nabla (k\nabla T)+S_{T}$$

where *u*, *υ*, and *ω* are the velocity of the molten material, *μ* is the viscosity of the molten material, *p* is the pressure, *T* is the temperature, *S_u_*, *S_v_*, and *S_w_* are the momentum equation source term, *S_T_* is the energy equation source term which contains contributions from radiation as well as any other volumetric heat sources, *V* is the velocity, *k* is the thermal conductivity, and *ρ* is the density.

In addition, we used the classic volume of fraction method (VOF) to track changes on the surface of the molten pool. The VOF method uses the function *F*(*x*,*y*,*x*) to represent the volume fraction of the fluid in the grid cell, which satisfies the following equation:

(6) $$\frac{\partial F}{\partial t}+u\frac{\partial F}{\partial x}+v\frac{\partial F}{\partial y}+w\frac{\partial F}{\partial z}=0$$

where *F* is the average volume fraction of fluid in the cell grid in the calculation domain. *F* = 1 indicates that the fluid volume fills the cell grid completely, *F* = 0 indicates that the fluid volume fraction is empty, and 0 < *F* < 1 indicates that both fluid and void interface are in the cell grid.

### 2.2. Heat Source

**Figure 1:**
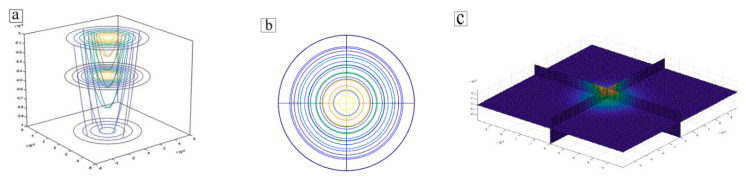
Heat source model. (**a**) The distribution of heat source energy. (**b**) The plane distribution of the heat source. (**c**) A three-dimensional slice of the heat source.

In the selective laser melting process, the laser heat source quickly scans the surface of the powder layer. The heat source model is shown in Figure 1. The laser beam has only a thermal effect. It is generally considered that the laser heat source is Gaussian distributed and there is a powder layer on the surface of the metal substrate. Therefore, the laser heat source model is established as follows:

(7) $$Q_{1}=\frac{\alpha A_{1}q_{1}}{\pi R^{2}\delta }\exp(-\alpha \frac{x^{2}+y^{2}}{R^{2}})\exp(-\frac{\left|z\right|}{\delta })\;|z|<\delta_{0}$$

(8) $$Q_{2}=\frac{\alpha A_{2}q_{2}}{\pi R^{2}}\exp(-\alpha \frac{x^{2}+y^{2}}{R^{2}})\;|z|=\delta_{0}$$

where *A*_1_ is laser absorption rate of powder, *A*_2_ is laser absorption rate of substrate, $q_{1}$ is the laser power irradiated on the powder layer, and $q_{2}$ is the laser power irradiated on the surface of the metal substrate. *α* is the heat source concentration factor, *R* is the laser radius, and *δ* is the laser penetration depth of the powder layer. $\delta_{0}$ is the powder layer thickness.

### 2.3. Model Computing Domain and Power Bed Model

**Figure 2:**
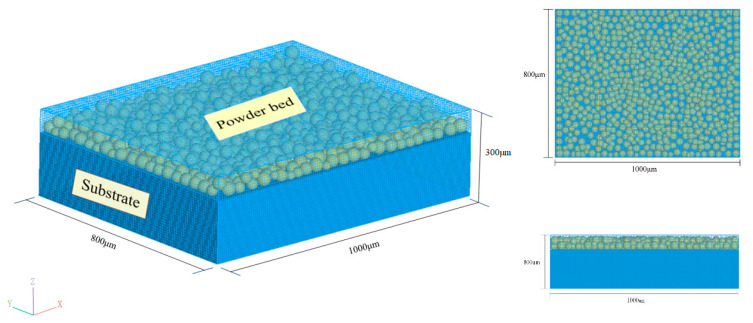
The computational domain of the 3D molten pool heat transfer simulation.

As shown in Figure 2, the calculation domain size is 1000 μm in the *x* direction, 800 μm in the *y* direction, and 300 μm in the *z* direction, and the substrate height is 250 μm. The minimum size of the grid is 6 μm, resulting in a total of 450,000 grids. Relative packing density was used to evaluate the quality of the powder bed. The particle size distribution of the powder was obtained by statistical analysis of the particle size of the pure molybdenum spherical powder. Relative packing density refers to the ratio of the volume of all powders in the powder bed to the physical volume of the powder layer space. In this paper, relative packing density reached 54%, which is roughly the same as the packing density reported previously [15] which did not exceed 60%, as shown below:

### 2.4. Boundary Conditions

The substrate and powder layer dissipate heat according to the following equation:

(9) $$q_{L}=h_{c}\left(T-T_{R}\right)-\sigma \varepsilon \left(T^{4}-T_{R}^{4}\right)+q_{evap}$$

(10) $$q_{evap}=\frac{0.82\Delta H^{*}}{\sqrt{2\pi \mathrm{MRT}}}P_{0}\exp\left(\frac{\Delta H^{*}\left(T-T_{V}\right)}{RTT_{V}}\right)$$

where *h_c_* is convection heat transfer coefficient, *T* is the surface temperature, *T_R_* is ambient temperature, ρ is the Stephen–Boltzmann constant, and its value is $5.67\times 10^{8}$ W/(m^2^K^4^). The first two terms on the right side of the equation correspond to heat convection and heat radiation, respectively. *q_evap_* is evaporative heat loss. When the surface temperature of the material exceeds the evaporation temperature, it will cause the metal vapor to escape, taking away a large amount of trace. The expression of metal vapor is reported by Karg [16]. R is the gas constant. *P*_0_ is atmospheric pressure. Δ${Η}^{*}$ is the effective evaporation enthalpy. M is the molar mass.

The driving force of the flow in the molten pool includes the buoyancy and the shear stress caused by the surface tension gradient, and the recoil pressure caused by the metal vapor on the surface of the molten pool must be considered here. The boundary control equation is expressed as follows:

(11) $$\gamma \left(T\right)=\gamma_{m}+\frac{d\gamma }{dT}\left(T-T_{m}\right)$$

(12) $$p_{vap}=p_{0}\cdot \exp\left(\frac{H_{v}M}{R}\left(\frac{1}{T_{V}}-\frac{1}{T_{}}\right)\right)$$

where $\gamma_{0}$ is the surface tension when the metal melts, *T_m_* is melting temperature, $\frac{d\gamma }{dT}$ is temperature coefficient of surface tension, *P*_0_ is atmospheric pressure, and $P_{vap}$ is metal vapor recoil pressure.

It is worth noting that argon gas is loaded as a protective gas through the subroutine, and the laser heat source, evaporation heat loss, and steam recoil pressure are also loaded through the subroutine.

### 2.5. Thermal Properties of Materials

**Figure 3:**
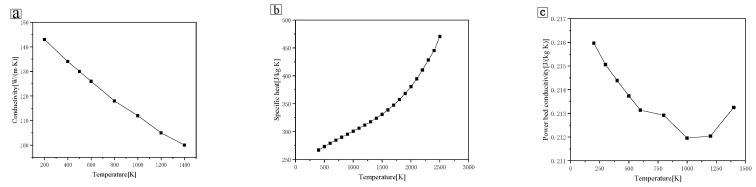
Temperature-dependent parameters of molybdenum alloy. (**a**) metal matrix conductivity; (**b**) specific heat; (**c**) thermal conductivity of powder bed.

The thermal properties of Mo material are shown in Figure 3. Considering that the thermal conductivity of the powder layer is much lower than that of the metal substrate, owing to the fact that the gap existing in the powder layer greatly reduces the heat transfer efficiency, the thermal conductivity of the powder layer was simplified according to the following formula [17,18]:

(13) $$\frac{k}{k_{f}}=(1-\sqrt{1-k_{\phi }})(1+\frac{k_{\phi }\cdot k_{r}}{k_{f}})+\sqrt{1-k_{\phi }}\left\{\frac{2}{1-\frac{k_{f}}{k_{s}}}\left[\frac{1}{1-\frac{k_{f}}{k_{s}}}\ln(\frac{k_{s}}{k_{f}})-1\right]+\frac{k_{r}}{k_{f}}\right\}$$

(14) $$k_{r}=4B\sigma T^{3}D_{p}$$

where $k_{\emptyset }$ is the porosity of the powder bed, $k_{f}$ is thermal conductivity of the shielding gas, $k_{s}$ is thermal conductivity of the metallic solid, and $k_{r}$ is the thermal conductivity caused by radiation among particles. $D_{P}$ is the average diameter of the granular powder, and B is the apparent coefficient, set here as 1/3.

The main parameters of Mo materials are shown in Table 1.

## 3. Selective Laser Melting Experiment

**Figure 4:**
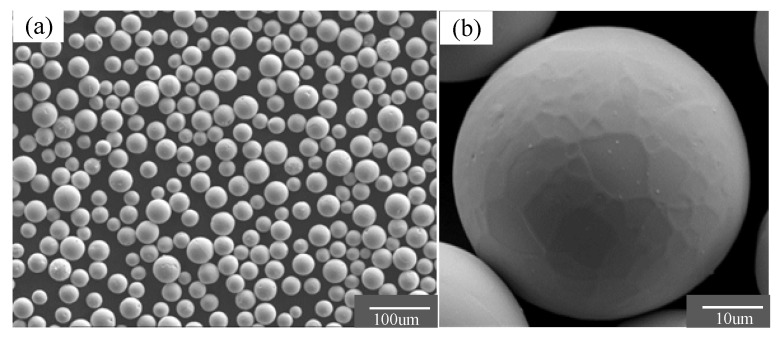
SEM images of spherical molybdenum powder. (**a**) ×200; (**b**) ×1000.

The experiment of printing pure molybdenum by the selective laser melting method was completed by BLT-S210 equipment (Bright Laser, Xi’an, China). The pure molybdenum with different scanning laser parameters was subjected to single track-forming experiments and the experiment was carried out under argon protective gas. As shown in Figure 4, spherical molybdenum powders (Star Technology Co., Ltd., Shenzhen, China) with purity of 99.98% were evaluated on the basis of SEM (Tescan-vega3).

## 4. Results and Discussion

### 4.1. Optical Microscope (OM) and Simulation Morphology Diagram of Single Track

In order to obtain the reasonable range of parameters on SLM forming pure molybdenum alloy, the single-pass melting experiment was carried out to reflect the over-melting and incomplete melting of the molten pool. So as to obtain the optimized range of laser power and scanning speed, the energy input per unit volume was used to evaluate the appropriate energy input range as shown in Table 2:

(15) $$\psi =\frac{2P}{hdv}$$

where *P* is the laser power, *h* is the powder layer thickness, *d* is the laser diameter, and *v* is scanning speed.

**Figure 5:**
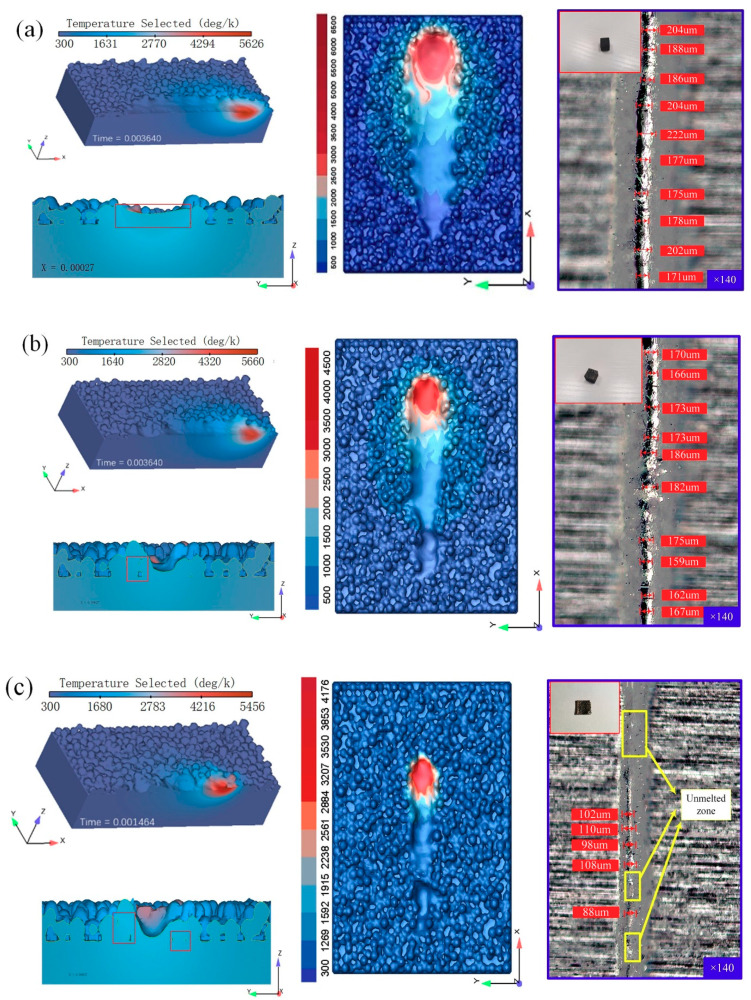
Melt pool morphology at different energy levels (single track of simulation, actual printing and final shape). (**a**) 4.0 × 10^11^ J/m^3^, P = 400 W, V = 400 mm/s; (**b**) 3.1 × 10^11^ J/m^3^, P = 310 W, V = 400 mm/s; (**c**) 1.6 × 10^11^ J/m^3^, P = 400 W, V = 1000 mm/s.

Figure 5 shows the morphology of the molten pool with different energy densities at 4.0 × 10^11^ J/m^3^, 3.1 × 10^11^ J/m^3^, and 1.6 × 10^11^ J/m^3^. To ensure that pure molybdenum with high melting point could get obtain heat to be melted, a lower scanning speed of 400 mm/s was selected. The selection of this speed value was also based on the experience from SLM tests [19,20,21]. It can be clearly seen from Figure 5a–c that as the laser energy density decreased, the melting channel became more and more uneven. Figure 5a shows the flattest melting channel, while the melting channel in Figure 5c is full of bumps and hollows. Additionally, during the initial stage of laser processing there was even a situation where the powder could not be melted, as shown in Figure 5c. As previously expected, the laser energy density had a huge impact on the morphology. With the same power in Figure 5a,c, when the scanning speed was accelerated, the melting channel in Figure 5c became discontinuous, which could also be proved by the OM pictures. Subsequently, Figure 5a shows the widest melt channel, while the width of melting channel in Figure 5c is roughly equivalent to the laser diameter. A too-narrow melting channel was not conducive to wetting with the surrounding powder, and also caused the overlapping rate to be low. The narrow melting channel easily stuck to the powder, resulting in an uneven melting channel. As a consequence, the porosity of the molded part was high. In fact, it was appropriate to keep the melt channel width at 1.5–2 times the laser diameter, which was consistent with the OM results shown in Figure 5a,b.

As shown in Figure 5a–c, the metal solidification plane in the same location after laser scanning was below the powder layer because of the porosity formed by the accumulation of powder particles in the initial stage. Due to the high laser energy density, the pores around the melting channel in Figure 5a are almost completely filled, and the melt transport in the metal melting pool is very uniform. This is ascribed to the low scanning speed and longer duration of time that the laser stayed on the surface of the powder layer. The molten powder had sufficient time to exchange heat with the surrounding powder, which was greatly beneficial to the compactness of the specimen. However, Figure 5b,c shows poor wettability with the surrounding powder particles, and the re-melting depth was not sufficient, meaning that the pores could not be completely filled through the molten pool flow marked by the red rectangle between the powder layers. In addition, as can be seen from Figure 5c, when the laser power and scanning speed were high, a sharper melt channel appeared owing to the lowest energy input. Therefore, maintaining a high energy input at not less than 3.1 × 10^11^ J/m^3^ was effective for forming materials with high melting points.

From observing the temperature distribution of the molten pool, it was not difficult to find that the isotherm of the head of the molten pool was dense, reflecting a large temperature gradient which was due to the low thermal conductivity of the un-melted powder particles with poor ability to transfer heat. On the contrary, the end of the molten pool showed a lower temperature gradient, which was due to the increased thermal conductivity of the material after solidification. This led to the appearance of a “droplet-shaped” molten pool. It can also be seen from Figure 5 that in the case of the lowest energy density, the alloy could not be formed and the printing process was terminated. At the other two energy densities, the alloy was completely formed.

**Figure 6:**
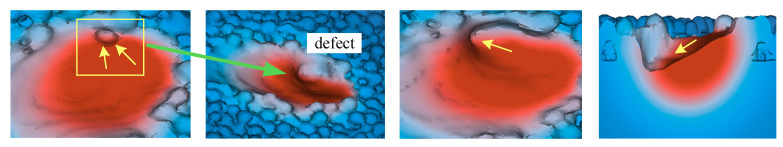
Schematic diagram of defects appearing at the edge of the melt channel with energy level 9 × 10^11^ J/m^3^, P = 450 W, and V = 200 mm/s.

According to Figure 6, when the power was increased and the energy density reached 9 × 10^11^ J/m^3^, the defects in the laser scanning process were clearly visible. The major cause for this phenomenon was the excessive energy per unit volume. Meanwhile, the powder was fully melted, the fluidity of the molten metal increased, and the existence time of the micro-molten pool grew. Under the action of surface tension, molten metal liquid flew into the adjacent forming melting channel, causing the current melting channel to be uneven. Therefore, the volume energy density (9 × 10^11^ J/m^3^) was too large for manufacturing the molybdenum alloy.

**Figure 7:**
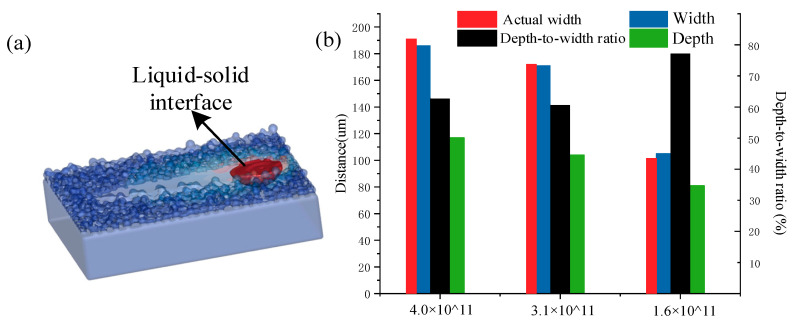
Molten pool data. (**a**) Molten pool shape. (**b**) Actual width of the molten pool obtained from the experiment and the width, depth, and depth-to-width ratio of the molten pool obtained from simulation.

The effect of laser energy density on the shape of the molten pool was also investigated quantitatively. Figure 7 shows the effect of the energy density on the width, depth, and depth-to-width ratio of the molten pool. The actual melt channel width was in agreement with the simulation results. As can be seen from the Figure 7b, with the increase of laser energy density, the depth of the molten pool increased from 81 μm to 117 μm and the width of the molten pool increased from 105 μm to 186 μm, but the depth-to-width ratio decreased from 77% to 62.9%. When the energy density was low, the width of the molten pool was close to the laser spot diameter and the depth of the molten pool was slightly shallower. It can be seen that the width of the molten pool in Figure 7b was more suitable, reaching a level 1.5–2 times the spot diameter with the exception of the final one where the energy density value reached 1.6 × 10^11^ J/m^3^. It was also found that when the scanning speed was 400 mm/s, simply increasing the laser power could not effectively increase the depth-to-width ratio of the molten pool. However, the influence of the scanning speed on the width of the molten pool was greater than on the depth of the molten pool (the melting width increased by 77%, and the melting depth increased by 44%) Furthermore, with the increased the scanning speed, the large growth of the depth-to-width ratio proved that the molten pool became deeper. This demonstrated that the flow of the molten pool became more unstable, causing the solidified metal melt channel to be rugged.

### 4.2. Melt Pool Structure and Powder Spatter over Time

**Figure 8:**
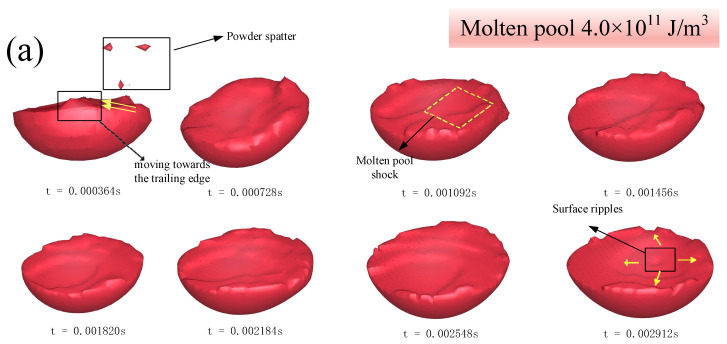

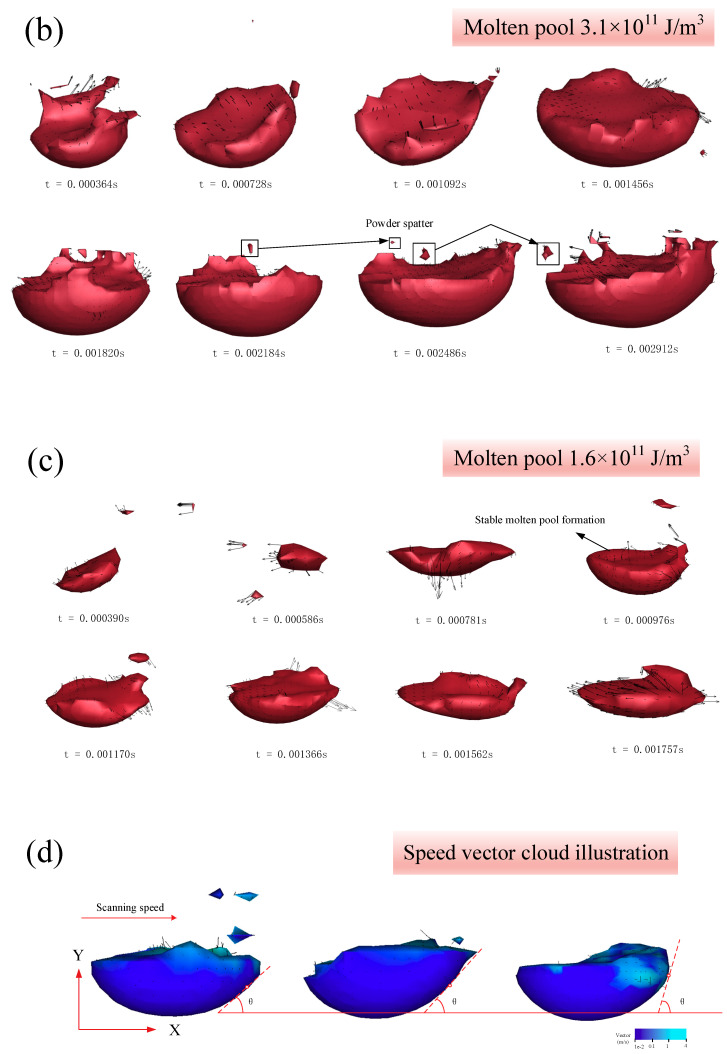
Macro view of the molten pool. (**a**) 4.0 × 10^11^ J/m^3^; (**b**) 3.1 × 10^11^ J/m^3^; (**c**) 1.6 × 10^11^ J/m^3^; and (**d**) speed vector cloud illustration of 4.0 × 10^11^ J/m^3^.

The shape of the molten pool with different laser energy density values was plotted in Figure 8, which shows the velocity distribution on the microscopic molten pool. Figure 8a shows the most uniform molten pool shape, except for a small amount of powder spatter at the initial stage. During the subsequent scanning process, the molten pool morphology had been kept in a stable state. Conversely, the powder spatter in the molten pool in Figure 8b was constantly present during the single-track scanning process. With the laser power decreasing, the molten pool flow became more and more unstable, and as the scanning speed increased, the molten pool splashing behavior became more and more intense. This was largely due to insufficient heat acting on the high melting point molybdenum powder. When the laser energy density was at the level of 4.0 × 10^11^ J/m^3^, a relatively stable molten pool shape appeared around 360 μs. However, Figure 8b shows that it appeared at around 728 μs, and in Figure 8c it appeared later at 1000 μs. Thus, higher energy input could make the molten pool reach its full shape sooner.

As observed from the morphology of three groups of molten pool changing with time, the powder spatter always appeared on the edge of the molten pool surface, where it was in contact with other spherical powders. The leading edge of molten pool underwent drastic boundary motion as the laser source moved forward. There are two reasons for the above phenomenon. On the one hand, although the evaporation temperature of the molybdenum alloy was very high, it could still be observed that the metal vapor recoil pressure appeared on the surface of the molten pool, resulting in a downward depression in the laser direct range, and the droplet was pushed out to the edge of molten pool. At the same time, the inclination angle of the leading molten pool increased, as shown in Figure 8d. The laser was directed at the front edge of the molten pool, resulting the metal droplets formed by the metal vapor recoil pressure being jetted backward, with the appearance of the keyhole phenomenon. On the other hand, insufficient laser energy caused the powder bed to fail to melt completely, and insufficient wetting with the surrounding powders resulted in the relatively stable molten pool morphology appearing later. For the these two reasons, the molten pool at lower energy density reached a relatively stable state after a long time and was coupled with turbulence, so the powder spatter phenomenon was more frequent.

### 4.3. Impact of Marangoni Flow on Defect Suppression

**Figure 9:**
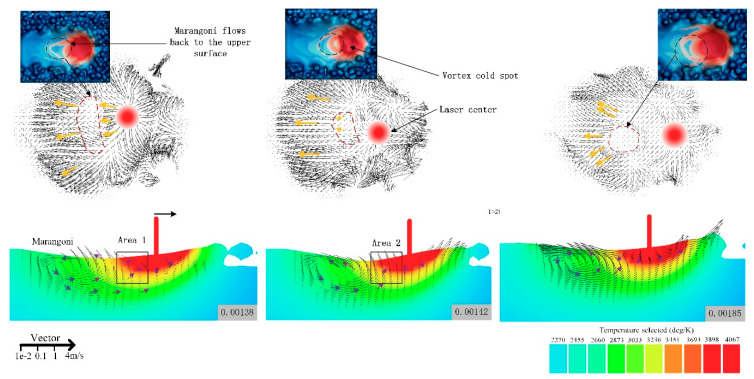
Vector diagram of molten pool at 0.00138 μs, 0.00142 μs, and 0.00185 μs.

Figure 9 shows the velocity field distribution at 0.00138 μs, 0.00142 μs, and 0.00185 μs, and the Marangoni flow effect can be clearly seen. The velocity vector field was formed by the vortex circulates counterclockwise from the back depression. After the laser was stopped, the center temperature decreased and the surface tension increased, overcoming the effect of the recoil force [22,23]. The melt velocity reversed suddenly. This led to the appearance of vortex cold spots as shown in the red line. The reflow helped accelerate the heat transfer and made molten pool larger and deeper. At the same time, this Marangoni flow contributed to the smoother melt channel and weakened the ‘depression’ caused by the metal vapor. As a result, this flow helped reduce the occurrence of porosity and increase the density of the metal. Adopting appropriate laser energy density input controlled the strength of the Marangoni flow and the stability of the molten pool, which was conducive to the smoothing of the molten channel and reduced the roughness.

The above phenomena were closely related to the laser interaction time. Therefore, exploring the laser interaction time was helpful for revealing the mechanism of selective laser melting on molybdenum alloy.

### 4.4. The Effect of Laser Interaction Time

The time that the laser stayed on the surface of the molybdenum material directly affected the existence time of the micro-molten pool and the quality of the melting channel, which could be evaluated by two significant parameters: melting time and evaporation time. Meanwhile, the droplet spreading/solidification competition mechanism was also an important factor affecting the molding quality, because the dominant spreading time was beneficial to defect suppression. Furthermore, both were related to the laser interaction time. Therefore, exploring the appropriate laser interaction time would help find the optimal working window for processing molybdenum alloys.

Melting time ($t_{melt}$) and evaporation time ($t_{vapor}$) were calculated as follows:

(16) $$T=\frac{AIR}{\sqrt{2\pi }k}\arctan\sqrt{\frac{8\alpha (t_{melt},t_{vapor})}{R^{2}}}$$

(17) $$I=\frac{2P}{\pi R^{2}}$$

(18) $$t_{d}=\frac{l_{g}}{v}$$

where *I* is the laser intensity, α is the thermal diffusion coefficient, $t_{d}$ is the laser residence time per unit length, *T* is melting point or evaporation point, and $l_{g}$ is the grid size. $t_{melt}$ and $t_{vapor}$ were evaluated under the various respective laser energy densities. *R* is the laser radius.

**Figure 10:**
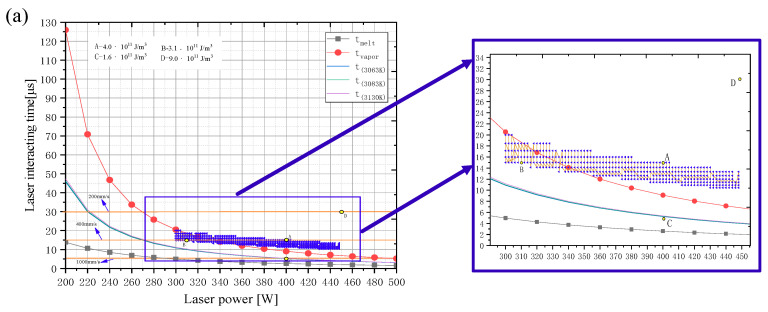

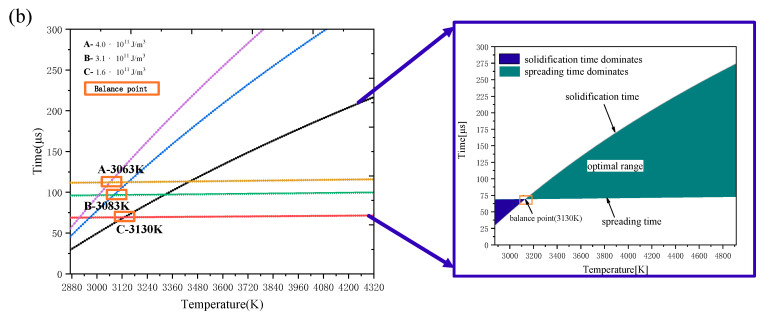
The relationship between laser interaction time and spread-solidification mechanism. (**a**) Laser residence time required to reach evaporation point and melting point at different energy densities. (**b**) Solidification time and spreading time calculated with three energy densities.

As shown in Figure 10a, the continuous laser radiation time to be maintained for a 6-μm grid length was calculated. The red line represents the laser residence time required for the molybdenum material to reach the evaporation point at various energy densities, and the black line represents the laser irradiation time to reach the melting point. The four yellow points refer to the time required under the various energy densities. It can be seen that when the power was 400 W and the scanning speed increased to 1000 mm/s, the radiation time was lower than the time to reach the evaporation point. This was close to the timeline required for the melting point, indicating that the powder did not have enough time to be fully melted, consistent with the shape of the melt channel in Figure 5c. For the point where the laser energy density was 4.0 × 10^11^ J/m^3^, the irradiation time was higher than that required for evaporation, which was also consistent with the macro behavior shown in Figure 5. However, when the input energy reached 9.0 × 10^11^ J/m^3^, the position shown was much higher than the red line of the evaporation point, demonstrating that ‘over-melting’ would occur and there would be a larger steam recoil pressure. Therefore, all phenomena indicated that the laser melting behavior was optimal when the energy density was between 4.0 × 10^11^ J/m^3^ and 3.1 × 10^11^ J/m^3^. The relationship between the energy density in this range (area drawn by the blue points) and the laser interaction time is plotted in Figure 10a.

In addition, the droplet spreading/solidification competition mechanism contained two significant time parameters: solidification time and spreading time [24,25].

(19) $$\tau_{solidification}=2\left(\frac{a^{2}}{3\alpha }\right)\ln\left(\frac{T_{}-T_{0}}{T_{f}-T_{0}}\right)$$

(20) $$\tau_{spread}=\sqrt{\left(\frac{\rho_{m}a^{3}}{\sigma }\right)}$$

where a is the droplet size, which depends on the size of the molten pool at three energy densities as shown in Table 3. $T_{f}$ is the solidus temperature. α is thermal diffusion coefficient. $\rho_{m}$ is the melting density.

The spreading action of droplets in the selective laser melting process was driven by capillary force and was also hindered by inertial force [26]. This competition mechanism was more closely related to the solidification time. With their combined action, the problem of spheroidization would be caused. Therefore, the spreading time and solidification time must be considered. It should be noted that pure molybdenum had the highest melting point, presenting a greater viscosity, and its fluidity was severely hindered. In addition to its high thermal conductivity and melting point, its solidification process was very rapid. If the laser speed moved too fast, the heat input was low, resulting in a low temperature. The solidification time was dominant, and the droplets were solidified before they spread. Therefore, the laser energy density could not be too low, otherwise the lower temperature of the droplet would lead to an advantage in the solidification process and the melting-solidification process could not be completed well. Reducing the scanning speed contributed to increased heat input and a slower solidification process, so that the droplets had enough time to spread. However, it should be noted that the above process was in an ideal situation. The actual droplet flow involved complex factors. When the heat input was too high, it also increased the droplet size “a” and led to a prolonged spreading time, which was not conducive to the molten pool being in a balanced position. In order to avoid the problem of spheroidization, the appropriate laser continuous irradiation time should be maintained to achieve the spreading time occupying the dominant position. In Figure 10b, the green area indicates that the spreading time was dominant, while the blue area indicates to the predominance of solidification time. As a result, the generation of defect voids was reduced, the wettability was improved, and the surface of the formed part was relatively flat.

When the spreading time was dominant, the spherical powder had to reach the temperature at which spreading time dominated. Thus, the molten droplet had enough time to spread. The dominant curves of spreading time under the three energies are shown in Figure 10a. It can be clearly seen that point ’C’ in Figure 10a was below the spreading time dominance curve. The melting channel under the energy density (1.6 × 10^11^ J/m^3^) was thus the most uneven. The area drawn by blue points in Figure 10a reached the requirements which had been proven. Consequently, as long as the laser energy density was in the green area, it was suitable for spreading time.

## 5. Conclusions

In this paper, the multi-field simulation of the selective laser melting process for the molybdenum alloy was carried out, the appropriate laser energy density region was explored, and some microscopic behaviors of molten pool were explained. The molybdenum alloy formed by the selective laser melting method was consistent with the simulation results. The following conclusions were drawn:

1. According to the simulation and experiments results of the pure molybdenum single track, it was concluded that keeping the energy density in the range of 3.1 × 10^11^–4.0 × 10^11^ J/m^3^ maintained appropriate melt channel depth and width. Otherwise, over-melting or insufficient melting would occur and layer-by-layer printing would cause the defects to be enlarged.
2. In the process of printing pure molybdenum, the occurrence of laser melting defects was related to molten pool convection and surface ripples. The Marangoni flow backflow helped to fuse the pores and inhibit the generation of defects, while higher energy input was likely to cause unsteady oscillations at the edge of the molten pool, where it was easy for defects to be produced.
3. The laser interaction time *t_melt_* and *t_vapor_* values were used to measure the time to reach the melting and evaporation points under each laser parameter in the simulation. Considering the solidification/spreading competition mechanism and the range of laser parameters at a suitable density, the processing window for the droplet to maintain better spreading behavior was obtained, which proved that the molten droplet could better complete the spreading behavior when the laser action time was higher than the equilibrium point. This provides a simulation theory guide from the perspective of laser interaction time for the future additive manufacturing of molybdenum alloys.

## Figures and Tables

**Table 1:** Molybdenum alloy and argon gas-related material properties taken from the material library.

Density ρ [kg/m^3^]	10.2·10^3^
Viscosity μ [Pa⋅s]	7.5·10^−3^
Surface tension σ [N⋅m]	2.27
Temperature coefficient $\frac{d\gamma }{dt}$ [(N⋅m)/T]	−0.0001
Absorption coefficient *A* [-]	0.34
Melting temperature *T_m_* [K]	2873
Evaporation temperature *T_v_* [K]	4912
Melting enthalpy *H_m_* [J/kg]	2.9·10^5^
Emissivity ε [-]	0.8
Convective heat transfer coefficient $h_{c}$ [W/m^2^]	80
Evaporation enthalpy *H_v_* [J/kg]	5.6·10^6^
Molar mass of metal vapor *M* [kg/mol]	0.096

The simulation process took about 50 h of CPU clock time to complete in a workstation with an Intel^®^ Xeon^®^ Gold 5218 CPU and 128 G RAM.

**Table 2:** The process parameters of selective laser melting (SLM).

Parameters	4.0 × 10^11^ J/m^3^	3.1 × 10^11^ J/m^3^	1.6 × 10^11^ J/m^3^	9.0 × 10^11^ J/m^3^
Laser power	400 W	310 W	400 W	450 W
Scanning speed	400 m/s	400 m/s	1000 m/s	200 m/s
Powder thickness	50 μm	50 μm	50 μm	50 μm
Laser diameter	100 μm	100 μm	100 μm	100 μm

**Table 3:** The droplet size depends on the simulation results.

Energy Densities	$a=R_{width}^{{}^{1/3}}D_{depth}^{2/3}(\mu m)$
4.0 × 10^11^ J/m^3^	136
3.1 × 10^11^ J/m^3^	125
1.6 × 10^11^ J/m^3^	100
